# A spatial-temporal description of the SARS-CoV-2 infections in Indonesia during the first six months of outbreak

**DOI:** 10.1371/journal.pone.0243703

**Published:** 2020-12-22

**Authors:** Dewi Nur Aisyah, Chyntia Aryanti Mayadewi, Haniena Diva, Zisis Kozlakidis, Siswanto, Wiku Adisasmito

**Affiliations:** 1 Indonesia One Health University Network, Depok, West Java, Indonesia; 2 Institute of Epidemiology and Health Care, University College London, London, United Kingdom; 3 World Health Organization, International Agency for Research on Cancer, Lyon, France; 4 National Institute of Health Research and Development, Jakarta, Indonesia; 5 Faculty of Public Health, Universitas Indonesia, Depok, West Java, Indonesia; 6 Indonesia National Task Force for the Acceleration of COVID-19, Jakarta, Indonesia; Jouf University, Kingdom of Saudi Arabia, SAUDI ARABIA

## Abstract

**Background:**

Since the first cases reported in Wuhan, China, in December 2019, the Severe Acute Respiratory Syndrome Coronavirus 2 (SARS-CoV-2) has spread worldwide. In Indonesia, the first case was reported in early March 2020, and the numbers of confirmed infections have been increasing until now. Efforts to contain the virus globally and in Indonesia are ongoing. This is the very first manuscript using a spatial-temporal model to describe the SARS-CoV-2 transmission in Indonesia, as well as providing a patient profile for all confirmed COVID-19 cases.

**Method:**

Data was collected from the official website of the Indonesia National Task Force for the Acceleration of COVID-19, from the period of 02 March 2020–02 August 2020. The data from RT-PCR confirmed, SARS-CoV-2 positive patients was categorized according to demographics, symptoms and comorbidities based on case categorization (confirmed, recovered, dead). The data collected provides granular and thorough information on time and geographical location for all 34 Provinces across Indonesia.

**Results:**

A cumulative total of 111,450 confirmed cases of were reported in Indonesia during the study period. Of those confirmed cases 67.79% (75,551/111,450) were shown as recovered and 4.83% (5,382/111,450) of them as died. Patients were mostly male (50.52%; 56,300/111,450) and adults aged 31 to 45 years old (29.73%; 33,132/111,450). Overall patient presentation symptoms of cough and fever, as well as chronic disease comorbidities were in line with previously published data from elsewhere in South-East Asia. The data reported here, shows that from the detection of the first confirmed case and within a short time period of 40 days, all the provinces of Indonesia were affected by COVID-19.

**Conclusions:**

This study is the first to provide detailed characteristics of the confirmed SARS-CoV-2 patients in Indonesia, including their demographic profile and COVID-19 presentation history. It used a spatial-temporal analysis to present the epidemic spread from the very beginning of the outbreak throughout all provinces in the country. The increase of new confirmed cases has been consistent during this time period for all provinces, with some demonstrating a sharp increase, in part due to the surge in national diagnostic capacity. This information delivers a ready resource that can be used for prediction modelling, and is utilized continuously by the current Indonesian Task Force in order to advise on potential implementation or removal of public distancing measures, and on potential availability of healthcare capacity in their efforts to ultimately manage the outbreak.

## Introduction

Since the first cases reported from Wuhan, China, in December 2019, the Severe Acute Respiratory Syndrome Coronavirus 2 (SARS-CoV-2) has spread worldwide. According to the World Health Organization (WHO) by August 23 2020, the number of total confirmed infected cases across the world stood at 23,057,288, while the total deaths reached 800,906 [1]. India and Indonesia, the two most populous countries in South-East Asia, have reported and continue to report large numbers of cases (3,044,940 and 151,498, respectively, as of August 23, 2020) [1]. In Indonesia, the first case was reported in March 02, 2020.

Efforts to contain the virus globally are ongoing. However, given the many uncertainties regarding the numbers of asymptomatic infections, as well as the rate of pathogen transmissibility, the effectiveness of these efforts is not adequately quantified yet. It has been demonstrated that the reproductive number of COVID-19 is higher compared to previous coronavirus outbreaks [2], and in addition a large proportion of infected cases seem to be asymptomatic [3]. Therefore, it is not surprising that the number of new confirmed cases and COVID-19-related deaths, are still rising in most countries, including in Indonesia.

In order to reduce the number of new cases, governments have relied on classical public health measures to curb the rate of spread of the epidemic. The primary goal of such public health measures (for example quarantine, social distancing, and community containment) has been consistently to prevent person-to-person transmission by separating and/or distancing people [4, 5]. Even though Indonesia implemented several control measures (including physical distancing, home quarantine, public messaging for the regular washing of hands and the wearing of masks in public, local travel restrictions, etc), there is still an upward trend in the number of new cases confirmed nationally. According to the WHO, the virus spread in Indonesia can be categorized as ‘community transmission’ [6], because of the inability to relate confirmed cases through chains of transmission for a large number of cases. Thus, understanding the transmission mechanisms and patterns forms a key question [7, 8].

A foundational first step in developing a strategy and predictive modelling for COVID-19 in Indonesia is to analyze the numbers of confirmed cases, along with their time occurrence [9]. This spatial-temporal reasoning model, is widely useful to obtain information about identifying high risk areas, supporting monitoring and prevention efforts [10], and to predict the epidemic’s trajectory [11].

To better understand the nature and spread of COVID-19 infection in Indonesia, we used spatio-temporal reasoning model to analyze the national data between March and August 2020. Data was collected from the Indonesia National Task Force for the Acceleration of COVID-19, from the period of March 2^nd^ 2020 to August 2^nfd^ 2020. The data provides granular information of COVID-19 surveillance in 34 Provinces across Indonesia and delivers a ready resource that can be used for future prediction modelling.

## Methods

### Study area

Geographically, Indonesia is in South-East Asia, lying between the Indian Ocean and the Pacific Ocean (lat: 5°00' N, lon: 120° 00' E). It is an archipelagic country comprising 5 major islands (Sumatera, Kalimantan, Java, Sulawesi, and Papua) and thousands of smaller islands. Indonesia is located adjacent to the equator line, and is a tropical region with 2 seasons: the rainy (October-March) and dry seasons (April-September).

Indonesia is administratively divided into 34 provinces and 514 cities and regencies, with independent local governments and parliamentary bodies. It has 10,138 public health centers (PUSKESMAS–primary healthcare facilities) and 2,902 hospitals (tertiary healthcare facilities) spread across these provinces, of which 132 hospitals are designated as national referral centres for the treatment of COVID-19 [12]. The total population of Indonesia stands at 267,663,435 (2018) with females representing 50% of the population [13], and the median age around 30 years [14].

### Data collection

Data for this study was obtained from the website of the Indonesian National Task Force for the Acceleration of COVID-19 Response (www.covid19.go.id). Data obtained included details of confirmed cases (i.e. demographics, presenting symptoms and comorbidities), and treatment outcomes (recovery or death) in all 34 provinces between 2nd March 2020 and 2nd August 2020. Use of the data, which was anonymized, aggregated, and at the population level was permitted by the Indonesian Ministry of Health under Regulation Number 45 (2014), Article 3, paragraph 1 and 2.

The Indonesian sampling and testing strategy aims to investigate all cases and clusters of COVID-19. The aim is for a minimum 90% of suspected cases to be isolated and to carry out a specimen collection in less than 48 hours since the appearance symptoms, so that secondary transmission is avoided as much as possible. The specimens should be sent to the referral laboratories and a result should be received within 72 hours, during which time individuals are advised to self-isolate. The outcomes of the epidemiological and laboratory investigations are required to be reported nationally on a daily basis to the Ministry of Health and the National COVID-19 Taskforce through dedicated data collection channels [15].

Samples are collected via nasopharyngeal (NP) or oropharyngeal (OP) swabs or sputum or serum, according to the Indonesian Ministry of Health protocols [15]. Mostly NP and OP swabs have been collected to date, for diagnostic testing performed by reverse transcriptase PCR (RT-PCR).

### Data analysis

The collected data was classified according to the recovered/death status to describe the patients’ characteristics. A univariate logistic regression was performed SPSS, version 20 (SPSS Inc., Chicago, IL, USA) to identify factors potentially associated with COVID-19 mortality. The adjusted odd ratios and 95% confidence intervals are shown for COVID-19 mortality risk factors based on sex, age group, and the geographical location (including only the 5 largest islands) [16]. The χ2 test was used to analyse the collected data about the impact of sex, region, and age group, as previously described [17].

Further descriptive presentation of the data was taken in 8 priority provinces, (Jakarta, West Java, East Java, Central Java, South Sulawesi, North Sulawesi, South Kalimantan, and Papua), in accordance with the recovered/death status, age group and sex characteristics.

For the spatio-temporal analysis, the number of new confirmed cases in each province were added into an xy axis graph and the amount of cases were differentiated by colours. To show the cumulative numbers of cases, the numbers were mapped by using available map customization application (https://mapchart.net/) [18]. The heat maps were created according to a window of 22 weeks follow-up.

## Results

A cumulative total of 111,450 confirmed cases of COVID-19 were reported in Indonesia between March 2^nd^ and August 2^nd^ 2020. 67.79% (75,551/111,450) of those confirmed cases were shown as recovered and 4.83% (5,382/111,450) of them as died (see Table 1). Among those reported as SARS-CoV-2 RT-PCR positive in Indonesia 50.52% were males (56,300/111,450) and adults aged 31 to 45 years old (29.73%; 33,132/111,450). There was no statistical significance observed in the sex ratio of confirmed cases (p = 0.339). However, sex was statistically significant for the recovered and death categories (p<0.001 in both cases), where greater relative proportions of females were recorded as recovered: the female group (68.05%; 35,352/51,948) compared to the male group (67.45%; 37,977/56,300). The death percentage is also greater in male (5.59%; 3,146/56,300) than female confirmed cases (4.09%; 2,127/51,948; p<0.001). Although adults aged 31–45 years old are shown to be more prone to be infected compared to other age groups, the elderly (≥ 60 years old) had the highest proportion of death (16.46%; 2,041/12,396; p<0.001) and lowest proportion of recovery across both genders (60.29%; 7,474/12,396; p<0.001), summarised in Table 2.

**Table 1:** Characteristics of total national demographics, symptoms and comorbidity based on case categorization (confirmed, recovered, death) in Indonesia from March 2^nd^–August 2^nd^ 2020.

Characteristics	Confirmed (n)	%	Active Cases (n)	% to total confirmed	Recovered (n)	% to total confirmed	Death (n)	% to total confirmed
All	111,450		30,517	27.38	75,551	67.79	5,382	4.83
Sex								
Male	56,300	50.52	15,178	26.96	37,977	67.45	3,146	5.59
Female	51,948	46.61	14,469	27.85	35,352	68.05	2,127	4.09
Missing	3,202	2.87	870	27.17	2,222	69.39	110	3.44
Age Group (y.o)								
0–5	2,507	2.25	733	29.24	1,723	68.73	51	2.03
6–18	7,197	6.46	1,969	27.36	5,169	71.82	59	0.82
19–30	24,557	22.03	7,210	29.36	17,108	69.67	239	0.97
31–45	33,132	29.73	9,359	28.25	22,987	69.38	786	2.37
46–59	26,000	23.33	6,722	25.85	17,238	66.30	2,039	7.84
≥60	12,396	11.12	2,881	23.24	7,474	60.29	2,041	16.46
Missing	1,777	1.59	1,643	92.46	1,207	67.92	68	3.83
Initial Symptoms								
Cough	2,871	2.58	70	2.44	2,400	83.59	401	13.97
History of fever	1,906	1.71	18	0.94	1,589	83.37	299	15.69
Fever	1,573	1.41	78	4.96	1,254	79.72	241	15.32
Difficulty to breathe	1,384	1.24	21	1.52	1,001	72.33	362	26.16
Fatigue	1,049	0.94	6	0.57	890	84.84	121	11.53
Cold	1,573	1.41	38	2.42	1,253	79.66	241	15.32
Sore throat	1,010	0.91	17	1.68	832	82.38	161	15.94
Headache	889	0.80	6	0.67	740	83.24	143	16.09
Nausea	769	0.69	6	0.78	640	83.22	123	15.99
Muscle cramp	613	0.55	6	0.98	500	81.57	107	17.46
Shivering	367	0.33	21	5.72	271	73.84	75	20.44
Diarrhoea	295	0.26	2	0.68	244	82.71	49	16.61
Stomach ache	304	0.27	4	1.32	248	81.58	52	17.11
Missing	104,150	93.45	30,336	29.13	68,988	67.24	4,825	4.63
Total Comorbidities								
None	5,246	4.71	140	2.67	4,927	93.92	178	3.39
One	1,166	1.05	16	1.37	786	67.41	186	15.95
Two	312	0.28	4	1.28	200	64.1	108	34.62
Three or more	113	0.1	0	0	55	48.67	58	51.33
Missing	104,791	94.03	30,357	28.97	69,582	66.4	4,852	4.63
Type of Comorbidity								
Hypertension	700	0.63	9	1.29	509	72.71	182	26.00
Diabetes mellitus	478	0.43	5	1.05	311	65.06	162	33.89
Heart disease	276	0.25	2	0.72	168	60.87	106	38.41
COPD	139	0.12	10	7.19	105	75.54	34	24.46
Other respiratory disease	89	0.08	2	2.25	63	70.79	24	26.97
CKD	83	0.07	0	0.00	40	48.19	43	51.81
TB	26	0.02	0	0.00	19	73.08	7	26.92
Pregnancy	68	0.06	6	8.82	56	82.35	3	4.41
Asthma	33	0.03	0	0.00	28	84.85	5	15.15
Cancer	21	0.02	0	0.00	14	66.67	7	33.33
Immunocompromise	21	0.02	0	0.00	14	66.67	7	33.33
Liver disease	28	0,03	0	0.00	20	71.43	8	28.57
Missing	104,791	94.03	30,357	28.97	69,582	66.4	4,852	4.63

**Table 2:** Risk factors (according to sex, age group and main island) for mortality of COVID-19 in Indonesia from March 2^nd^–August 2^nd^2020.

Risk Factors	Confirmed (n)	%	Death (n)	% death to total confirmed	Recovered (n)	% recovered to total confirmed	Univariable OR (Death)	Univariable OR (Recovered)
All	111,450		5,382	4.83	75,551	67.79		
Sex								
Female	51,948	46.61	2,127	4.09	35,352	68.05	1	1
Male	56,300	50.52	3,146	5.59	37,977	67.45	**1.376 (1.300, 1.457)**	**0.727 (0.686, 0.769)**
Age Group (y.o)								
0–5	2,507	2.25	51	2.03	1,723	68.73	1	1
6–18	7,197	6.46	59	0.82	5,169	71.82	**0.386 (0.264, 0.563)**	**2.593 (1.776, 3.787)**
19–30	24,557	22.03	239	0.97	17,108	69.67	**0.472 (0.347, 0.641)**	**2.119 (1.560, 2.878)**
31–45	33,132	29.73	786	2.37	22,987	69.38	1.155 (0.867, 1.540)	0.866 (0.649, 1.154)
46–59	26,000	23.33	2,039	7.84	17,238	66.30	**3.996 (3.013, 5.299)**	**0.250 (0.189, 0.332)**
≥60	12,396	11.12	2,041	16.46	7,474	60.29	**9.226 (6.954, 12.241)**	**0.108 (0.082, 0.144)**
Main Island								
Maluku & Papua Islands	6,266	5.62	111	1,77	3,493	55.75	1	1
Sulawesi Island	14,837	13.31	527	3,55	10,369	69.89	**1.599 (1.299, 1.970)**	**0.625 (0.508, 0.770)**
Kalimantan Island	10,156	9.11	436	4,29	6,721	66.18	**2.041 (1.651, 2.524)**	**0.490 (0.396, 0.606)**
Sumatera Island	10,785	9.68	498	4,62	6,070	56.28	**2.582 (2.093, 3.185)**	**0.387 (0.314, 0.478)**
Java & Bali Islands	69,396	62.27	3,810	5,49	48,898	70.46	**2.452 (2.024, 2.970)**	**0.408 (0.337, 0.494)**
Month								
March	868	0.78	148	17.05	706	81.34	1	1
April	8,444	7.58	707	8.37	7,577	89.73	**0.445 (0.367, 0.540)**	**2.247 (1.852, 2.726)**
May	16,251	14.58	967	5.95	14,823	91.21	**0.311 (0.258, 0.376)**	**3.213 (2.661, 2.881)**
June	29,795	26.73	1,694	5.69	25,882	86.87	**0.312 (0.260, 0.375)**	**3.203 (2.665, 3.849)**
July	52,371	46.99	1,700	3.25	25,839	49.34	**0.314 (0.261, 0.377)**	**3.186 (2.651, 3.929)**
August	3,104	2.79	68	2.19	161	5.19	**2.015 (1.442, 2.815)**	**0.496 (0.355, 0.693)**
Total Comorbidities								
None	5,246	4.71	178	3.39	4,927	93.92	1	1
One	1,166	1.05	186	15.95	786	67.41	**6.550 (5.263, 8.153)**	**0.153 (0.123, 0.190)**
Two	312	0.28	108	34.62	200	64.1	**14.947 (11.322, 19.732)**	**0.067 (0.051, 0.088)**
Three or more	113	0.1	58	51.33	55	48.67	**29.190 (19.605, 43.461)**	**0.034 (0.023, 0.051)**
Type of Comorbidity								
Hypertension	700	0.63	182	26.00	509	72.71	**6.034 (4.922, 7.398)**	**0.166 (0.135, 0.203)**
Diabetes mellitus	478	0.43	162	33.89	311	65.06	**8.273 (6.647, 10.296)**	**0.121 (0.97, 0.150)**
Heart disease	276	0.25	106	38.41	168	60.87	**9.029 (6.936, 11.754)**	**0.111 (0.85, 0.1444)**
COPD	139	0.12	34	24.46	105	75.54	**4.037 (2.710, 6.014)**	**0.248 (0.166, 0.369)**
Other respiratory disease	89	0.08	24	26.97	63	70.79	**3.581 (2.212, 5.798)**	**0.279 (0.172, 0.452)**
CKD	83	0.07	43	51.81	40	48.19	**13.745 (8.842, 21.366)**	**0.073 (0.047, 0.113)**
TB	26	0.02	7	26.92	19	73.08	**3.389 (1.416, 8.110)**	**0.295 (0.123, 0.706)**
Pregnancy	68	0.06	3	4.41	56	82.35	**1.274 (0.546, 2.972)**	**0.785 (0.337, 1.831)**
Asthma	33	0.03	5	15.15	28	84.85	**1.642 (0.631, 4.275)**	**0.609 (0.234, 1.586)**
Cancer	21	0.02	7	33.33	14	66.67	**5.993 (2.407, 14.918)**	**0.167 (0.067, 0.415)**
Immunocompromise	21	0.02	7	33.33	14	66.67	**6.001 (2.411, 14.939)**	**0.167 (0.067, 0.415)**
Liver disease	28	0.03	8	28.57	20	71.43	**4.806 (2.106, 10.969)**	**0.208 (0.091, 0.475)**

The most common initial symptoms reported as occurring among the confirmed infected population were cough (2.58%; 2,871/111,450), followed by a history of fever (1.71%; 1,906/111,450) and persistent fever (1.41%; 1,573/111,450). Not all of the patients suffered from difficulty to breathe (1.24%; 1,384/111,450). A number of confirmed cases also reported a clinical history of hypertension (0.63%; 700/111,450) and diabetes mellitus (0.43%; 478/111,450). The major comorbidities found among patients who were registered as dead included kidney disease (51.81; 43/83), heart disease (38.41%; 106/168), and immunocompromised patients (33.33%; 7/14). However, caution needs to be exercised when interpreting the above data, as the records have variable levels of completion for the initial symptoms categories and as such could not support further statistical analyses. The very high level of incomplete data (in some cases close to 90%, Table 2) and the high variability of completion between different comorbidity categories has prevented the further analyses of comorbidities.

The 5 provinces with the highest number of COVID-19 confirmed cases (as a % of the national total) were East Java (20.19%; 22,504/111,450), DKI Jakarta (19.87%; 22,144/111,450), Central Java (8.73%; 9,732/111,450), South Sulawesi (8.66%; 9,647/111,450), and West Java (5.96%; 6,637/111,450). These five provinces contributed over than 60% of the national cases. On the other hand, based on the reported case incidence, the top 5 provinces were DKI Jakarta (204.16 per 100,000 population), South Kalimantan (153.91 per 100,000 population), South Maluku (119.05 per 100,000 population), Gorontalo (108.75 per 100,000 population), and South Sulawesi (102.33 per 100,000 population). The recorded incidence in Jakarta as compared to the other main islands was much higher and statistically significant across all case categories (confirmed, recovered, death; p<001 for all).

**Fig 1:**
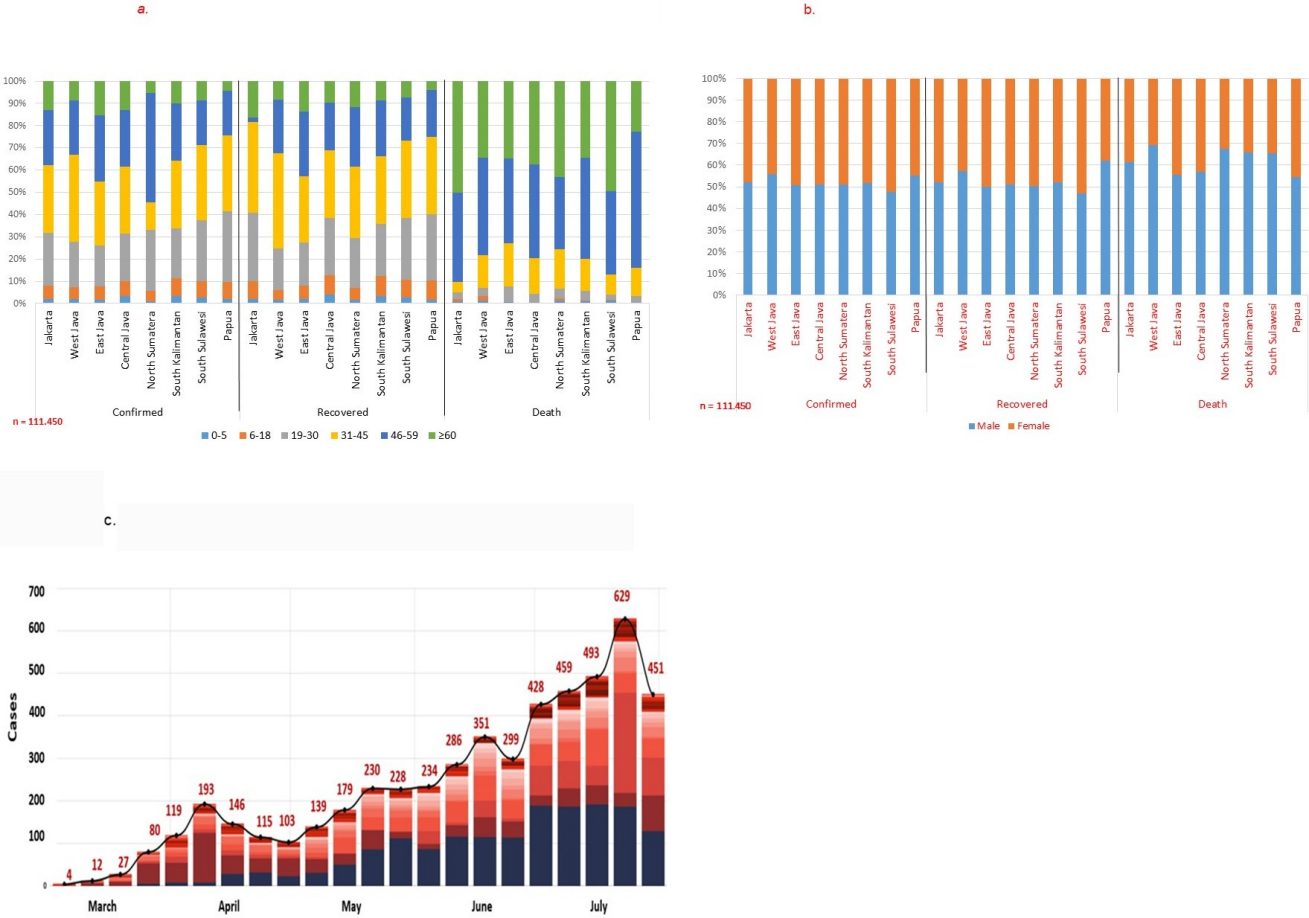
**a.** Classification of status according to age group in 8 priority provinces in Indonesia from March 2^nd^–August 2^nd^ 2020. **b.** Classification of patients’ status according to sex in 8 priority provinces in Indonesia from March 2^nd^–August 2^nd^ 2020. **c.** Incidence of death cases by week due to COVID-19 in Indonesia from March 2^nd^–August 2^nd^ 2020.

The characteristics of patients in Jakarta, West Java, East Java, Central Java, North Sumatera, South Kalimantan, South Sulawesi, and Papua for age group, sex, and recovered/death status are shown in Fig 1A and 1B, as these provinces had the highest reported COVID-19 incidence. The overall weekly related reporting of cases and deaths is shown in Fig 1C.

**Fig 2:**
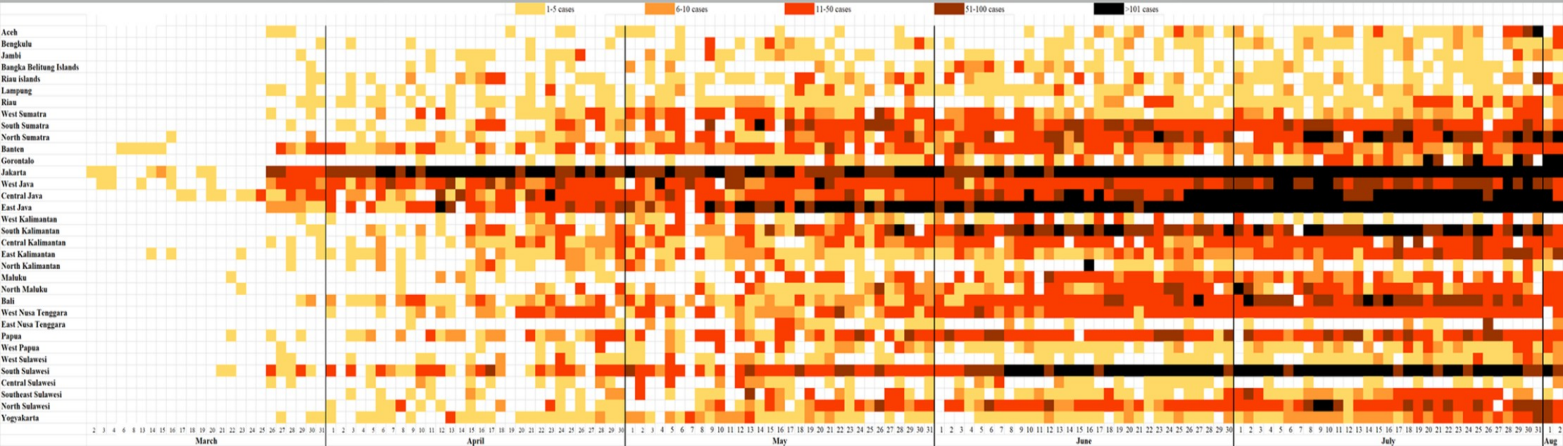
New confirmed cases per day in 34 provinces in Indonesia from March 2^nd^–August 2^nd^ 2020.

**Fig 3:**
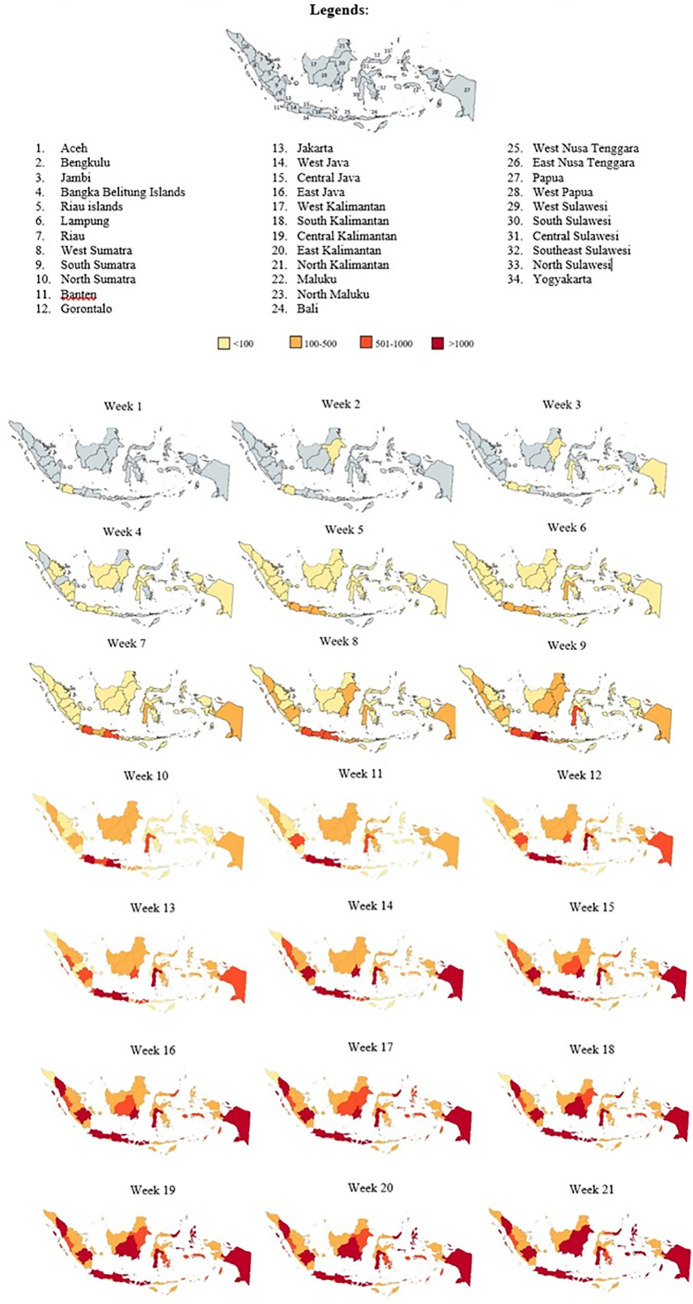
Distribution map of Covid-19 cumulative cases in Indonesia from March 2^nd^–August 2^nd^ 2020.

The first two RT-PCR confirmed cases of SARS-CoV-2 were identified on March 02, 2020 in Jakarta. The third positive confirmed case was found in West Java on March 03 and another case in Banten on March 06, 2020. The spatial-temporal results show that all provinces in Indonesia experienced confirmed COVID-19 cases (Figs 2 and 3) within 41 days since the first confirmed case was reported (March 2nd to April 10th 2020). Generally, Jakarta reported consistently the highest number of new confirmed cases per day (>100 per day). Even though the date of onset for the different outbreaks was different among different provinces (Fig 2), once a case was confirmed, new cases followed immediately afterwards.

**Fig 4:**
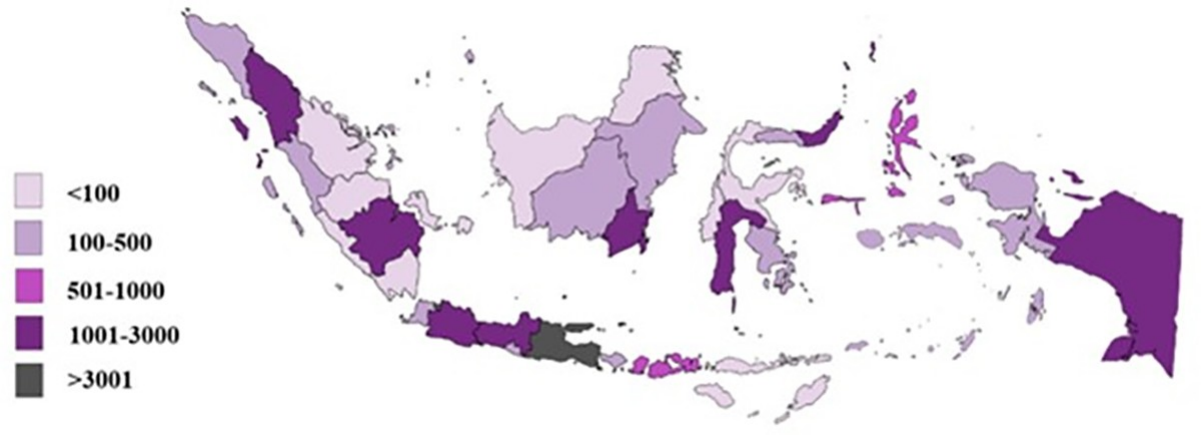
Distribution map of COVID-19 cases in Indonesia, August 2^nd^ 2020.

The heat map (Fig 3) shows the development of transmission in the 34 provinces across Indonesia within this period of approximately 6 months (5 months and 2 weeks). During the first week, the confirmed cases were concentrated only in West Java, Jakarta, and Banten. The following week, new confirmed cases were reported in East Kalimantan, and a few days later the spread of new infections was increasing rapidly both in the western and eastern part of Indonesia, adding 18 new provinces with reported cases. By the sixth week, confirmed cases were noted across all provinces. Within the last 2 weeks of the period studied, cumulative cases of >100 confirmed cases had occurred in several provinces, leaving Jakarta as the most infected area with >1000 cases by the end of the last week (Fig 4).

## Discussion

Overall, our study shows that the RT-PCR confirmed SARS-CoV-2 infections in Indonesia were more prevalent in males with age range between 31–45 years old, most presenting with self-reported fever and cough symptoms, and having a prior clinical history of hypertension and/or of diabetes mellitus. The highest increase in the absolute amount of new confirmed cases was shown in Jakarta, West Java, Central Java, East Java, and South Sulawesi; while other provinces showed a comparatively modest reporting of new confirmed cases every day. By the sixth week of follow-up all provinces in Indonesia were affected by confirmed COVID-19 cases. Similar transmission patterns were seen in 5 major cities in China, not geographically adjacent to Wuhan [19].

The characteristics of Indonesian COVID-19 patients were similar to those reported for China and Italy [20–22], with cough and fever/history of fever being the most frequent symptoms [20, 23] and within similar age groups [23, 24]. Males were more prevalent to a worse clinical outcome as compared to females as in other studies from China, Italy and the USA [20, 25, 26]. The elderly population of above 60 years old (19.8%; 326/1,647) showed the highest mortality, in line with observations in other countries [20–22]. Thus, current observations in Indonesia are aligned with previously published observations [27] and show similar patient profiles from other parts of South-East Asia [28–31]. Furthermore, as Indonesia reports one of the highest chronic kidney disease incidences in South-East Asia, affecting over 8% of the over 65 population group, this is likely to further compound the observed mortality rate in that population group as a linked underlying health condition [32].

The COVID-19 vulnerable groups definition includes elderly people, people who are ill and have comorbidities, homeless or under-housed people, as well as people who might struggle economically and psychologically [33]. Children and younger adults represent the smallest proportion of confirmed cases in Indonesia, in line with national reports elsewhere [34, 35]. As this is an inclusive national surveillance system, there is no particular systemic bias for the exclusion of particular vulnerable groups, children or bias in the recording of cases, other than the latter are probably asymptomatic or less symptomatic than other population groups. However, this hypothesis would still need to be verified clinically by the purposive sampling of this age group.

The increase in confirmed cases also reflects the increased availability of testing nationally, as the Indonesian Government allowed both governmental and non-governmental laboratories to perform confirmatory RT-PCR analyses, increasing the national diagnostic capacity [36–38]. By April 29^th^ 2020, there were in total 89 laboratories that have been appointed to examine the suspected patient samples, consisting of 48 hospital laboratories, 15 university laboratories, 18 Ministry of Health laboratories, and 3 from the Directorate of Livestock [36].

The Governor of Jakarta enacted Large Scale Social Distancing starting on April 10^th^ 2020 (40 days after the first confirmed COVID-19 case was reported). This initiative was followed by West Java’s Governor applying the same regulations in satellite cities near Jakarta. The effectiveness of the timing as well as the implementation of these regulations should be evaluated at a later point, however it can be said that they eventually (within 3–4 weeks) led to the plateau of the reported transmission rate within these cities. These measures were coupled with individual-level preventive and hygiene actions strongly advised by the Indonesian Government [39].

The strength of our study is that the data was collected centrally by the Indonesia National Task Force for the Acceleration of COVID-19. Therefore, this made the uniform in the way of reporting, and able to cover thoroughly the entire national area of Indonesia. However, this study has certain limitations. The very high level of incomplete data (in some cases close to 90%) and the high variability of completion between different comorbidity categories has prevented the further analyses of comorbidities. The level of completion (Table 2) is an aspect currently addressed by the Indonesian authorities. Furthermore, there were delays in the reporting of data, either due to the lack of staff confidence in the initial handling of the reports or more likely because of the overwhelmed data management structure at the local levels of administration. Furthermore, the data was not always complete, and did not provide patient’s history of social contact, while symptoms, such as cough and prior fever episodes were self-reported, making them prone to memory bias. As Indonesia is a very active transit hub in South- East Asia, there is a likelihood that a number of the confirmed COVID-19 cases might be imported from other nations regionally, however the type of data collected so far does not allow to differentiate local transmission events vs imported seeding from other areas.

## Conclusion

This study is the first to provide detailed characteristics of the laboratory confirmed COVID-19 patients in Indonesia and used a spatial-temporal analysis to present the transmission pattern from the very beginning of the outbreak in Indonesia on March 2^nd^ 2020 to August 2^nd^ 2020. The demographic profile of confirmed infected patients reported in this manuscript is in keeping with the confirmed patient profiles from other parts of South-East Asia. As in other neighbouring geographical areas (Vietnam, Thailand and Malaysia) the first confirmed cases were directly related to travellers from other infection hotspots. In the case of Indonesia, it is thought to have arrived from travellers returning from an affected area in Malaysia, as reported widely in the press [40]. However, without genomic or further laboratory information this remains an informed conjecture.

The data reported here, demonstrates that all the provinces of Indonesia were affected by COVID-19 within a short time period. The incidence on Java island is not surprising given its very high population density and population movement. The increase of new confirmed cases has been consistent during this time period for all provinces, in part due to the surge in national diagnostic capacity. This information is utilized continuously by the current Indonesian Task Force in their efforts to ultimately manage the outbreak.
